# Cyclin-Dependent Kinase 4/6 Inhibitors Combined with Radiotherapy in Curative Breast Cancer Patients Induced Pneumonitis: A Case Report

**DOI:** 10.3390/life15050709

**Published:** 2025-04-27

**Authors:** Pei-Yu Hou

**Affiliations:** 1 Department of Radiation Oncology, Far Eastern Memorial Hospital, New Taipei City 220216, Taiwan; jcgv03@mail.femh.org.tw; 2 Department of Computer Science and Engineering, Yuan Ze University, Taoyuan 320315, Taiwan

**Keywords:** breast cancer, radiation therapy, cyclin-dependent kinase 4/6 inhibitors, pneumonitis

## Abstract

Background: The role of CDK4/6 inhibitors (CDK4/6i) has expanded from the treatment of advanced breast cancer to early-stage disease, as recent studies have demonstrated their therapeutic benefits. However, evidence regarding the safety of combining CDK4/6i with adjuvant radiation therapy (RT) in a curative setting remains limited. This study aims to present clinical experiences of pulmonary toxicity following the combined use of adjuvant RT and CDK4/6i. Case presentation: We report a case of an Asian female with left breast cancer who underwent a modified radical mastectomy followed by adjuvant chemotherapy, RT, endocrine therapy, and CDK4/6i (abemaciclib) treatment. Cancer therapy-induced grade 2 pneumonitis was impressed by clinical signs and image findings. A 57-year-old postmenopausal woman was diagnosed with left breast invasive lobular carcinoma, hormone receptor–positive, human epidermal growth factor receptor 2–negative (HR+/HER2−), K67 index of 5–10%, and classified as pT3N3aM0 (stage IIIC). She received adjuvant chemotherapy with FEC followed by docetaxel, endocrine therapy with letrozole, and adjuvant RT of 50.4 Gy in 28 fractions to the left chest wall and regional nodal irradiation. Abemaciclib was initiated after completing RT. Treatment-related pneumonitis developed five months after RT and abemaciclib use. Conclusions: In breast cancer patients receiving a combination of RT and CDK4/6i as curative adjuvant treatment, pulmonary toxicity is a concern and requires careful monitoring, particularly in Asian populations.

## 1. Introduction

In recent years, significant advancements have been made in novel systemic treatments for breast cancer. One of these new approaches is the use of cyclin-dependent kinase 4/6 inhibitors (CDK4/6i), which are small-molecule inhibitors targeting CDK4 and CDK6. These inhibitors help overcome resistance to hormonal blockades in advanced breast cancer. The most commonly used CDK4/6i include ribociclib, palbociclib, and abemaciclib. The combination of CDK4/6i with endocrine therapy has significantly improved progression-free survival (PFS) and overall survival (OS) in patients with hormone receptor (HR)-positive, human epidermal growth factor receptor 2 (HER2)-negative advanced breast cancer. Several phase III randomized controlled trials (RCTs) have demonstrated their efficacy as a first-line treatment for patients with advanced or metastatic breast cancer [1,2,3,4,5].

In early-stage breast cancer, the monarchE trial established the invasive disease-free survival (iDFS) benefit of the addition of two years for adjuvant abemaciclib to endocrine therapy in HR-positive, HER2-negative patients with node-positive disease and a high risk of recurrence, defined as ≥four pathological involved lymph nodes (LNs) or one to three LNs and either tumor size ≥ 5 cm or grade 3 [6,7]. The additional two-year abemaciclib to endocrine therapy had reduced the absolute risk of disease recurrence at four years by 6.4% (85.8% in the abemaciclib plus endocrine therapy group vs. 79.4% in the endocrine therapy alone group, hazard ratio (HR) 0.664, *p* < 0.0001) [8]. Additionally, the NATALEE trial demonstrated that the addition of three years of ribociclib combined with an aromatase inhibitor (AI) significantly prolonged iDFS in patients with HR-positive, HER2-negative stage II or III early breast cancer [9]. The iDFS benefit at three years was 3.3% (90.4% with ribociclib plus AI and 87.1% with AI alone, HR 0.75, *p* = 0.003). As a result, the role of CDK4/6i has expanded from the treatment of advanced breast cancer to early-stage disease and is increasingly being used in curative treatment settings. According to National Comprehensive Cancer Network clinical practice guidelines in 2025, two years of adjuvant abemaciclib in combination with endocrine therapy (Tamoxifen or AI), or three years of adjuvant ribociclib combined with AI is recommended for eligible HR-positive, HER2-negative high-risk patients.

Radiation therapy (RT) is a key adjuvant treatment for breast cancer, particularly following breast-conserving surgery in patients with early-stage disease. Radiation-induced pneumonitis is a recognized toxicity associated with chest irradiation. Symptoms such as shortness of breath or a non-productive cough typically manifest within one to six months after treatment. The diagnosis of therapy-related pneumonitis requires the exclusion of other potential etiologies and should be based on a comprehensive evaluation of clinical symptoms, temporal association with treatment, and radiological findings. Corticosteroids remain the mainstay of treatment for symptomatic patients.

Previous studies evaluating the safety of combining CDK4/6i with RT have primarily focused on patients with advanced or metastatic breast cancer, where RT is typically administered at relatively low doses for palliative purposes [10,11,12,13,14]. These studies have reported acceptable toxicity profiles when CDK4/6i are combined with palliative RT, with the most adverse effects being hematologic, gastrointestinal (GI), or skin related. However, one case series documented severe grade 5 pneumonitis [14]. Although recent literature and clinical guidelines increasingly support the use of CDK4/6i in the treatment of early-stage breast cancer, there is a lack of evidence regarding the safety of combining these agents with adjuvant RT, which typically involves moderate to high radiation doses. It remains unclear whether this combination increases the severity or alters the pattern of toxicity. Here, we report a case of an Asian female who developed pneumonitis after receiving adjuvant chest RT and CDK4/6i in a curative setting. Through this case, we aim to contribute clinical insights into the safety of combining RT with CDK4/6i in early-stage breast cancer.

## 2. Case Presentation

### 2.1. Patient Population

We retrospectively reviewed the patient with breast cancer, who was receiving adjuvant RT and CDK4/6i at Far Eastern Memorial Hospital (FEMH) in 2024 and reported the associated cancer treatment-related toxicity. The severity of toxicity was classified according to Common Terminology Criteria for Adverse Events (CTCAE) v5. This study was approved by the Human Experimentation Committee of Far Eastern Memorial Hospital (FEMH No.: 114014-C).

### 2.2. RT Treatment Plan

Computed tomography (CT) simulation with a 2.5 mm slice thickness (Discovery CT590 RT, GE Healthcare, Chicago, IL, USA) was performed for RT treatment planning to delineate the target volume and adjacent OARs. The patients were placed in the supine position and allowed to breathe freely during CT simulation. The clinical tumor volume (CTV) included the chest wall (CW), supraclavicular and infraclavicular regions, any part of the axillary bed at risk, and internal mammary region for regional nodal irradiation (RNI). The planning target volume (PTV) was defined as the CTV plus a 5–6 mm margin to allow for setup errors. Daily image guidance with CT was used to define the anatomy and internal soft tissue position. The RT prescription was a conventional dose of 45–50.4 Gy in 25–28 fractions with a daily fraction to the chest wall and RNI.

### 2.3. RT Techniques and Dosimetry Evaluation

Helical tomotherapy (HT) was planned with the aim of accomplishing better homogeneity and conformity of target coverage while sparing adjacent normal organs to minimize exposure to the heart, lungs, and other organs at risk (OARs). A dose–volume histogram (DVH) was used to evaluate the radiation dose constraints of the target volumes and OARs. The following criteria were required: at least 100% of the CTV volume was to receive 100% of the prescription dose, at least 95% of the PTV volume was to receive 95–100% of the prescription dose, and the maximal dose to the PTV region should be less than 110% of the prescription dose. The constraints of OARs were a mean lung dose ≤ 20 Gy, V20 ≤ 35%, a mean heart dose ≤ 20 Gy, V25 < 10%, a maximal spinal cord dose < 45 Gy, and as low a dose as possible to the contralateral breast, with the usual requirements for other normal organs.

A tomotherapy Hi Art Planning system (version 5.1.3, Tomotherapy, Inc., Madison, WI, USA) was used to plan HT, which was delivered by using a Tomotherapy^®^ Hi-Art or HD system (Tomotherapy^®^; Accuray Inc., Madison, WI, USA).

## 3. Case

A 57-year-old post-menopausal woman had an underlying disease of dyslipidemia under medical control. She did not have a history of smoking or any pre-existing pulmonary disease. She had been diagnosed with left breast invasive lobular carcinoma, Grade 2, ER-positive (100%), PR-positive (100%), HER-2-negative (Score 0), K67 index of 5–10%. She underwent left modified radical mastectomy with tissue expander placement, and the pathological staging was pT3N3aM0 (stage IIIC). She completed adjuvant chemotherapy, consisting of four cycles of fluorouracil/epirubicin/cyclophosphamide (FEC), followed by four cycles of docetaxel, and then initiated endocrine therapy with letrozole at a daily dose of 2.5 mg. After completing chemotherapy, she received adjuvant RT with 50.4 Gy in 28 fractions to the left CW and 45 Gy in 25 fractions to the RNI. RT was delivered using HT. The radiation dose to OARs for the lungs was as follows: the ipsilateral lung received a mean dose of 11.9 Gy, with V20 of 21.9%, V10 of 38%, and V5 of 62%. For the whole lung, the mean dose was 7.5 Gy, with V20 of 10.3%, V10 of 18.6%, and V5 of 36.2%.

**Figure 1:**
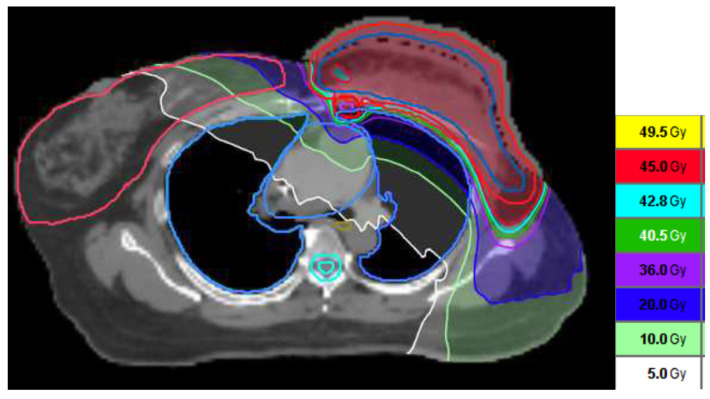
The dose distribution of RT planning.

The dose distribution of the initial 45 Gy covering the CW and RNI is shown in Figure 1. The tissue expander had ruptured before referring for RT preparation. The left CW CTV had covered the whole expander, as shown in Figure 1.

**Figure 2:**
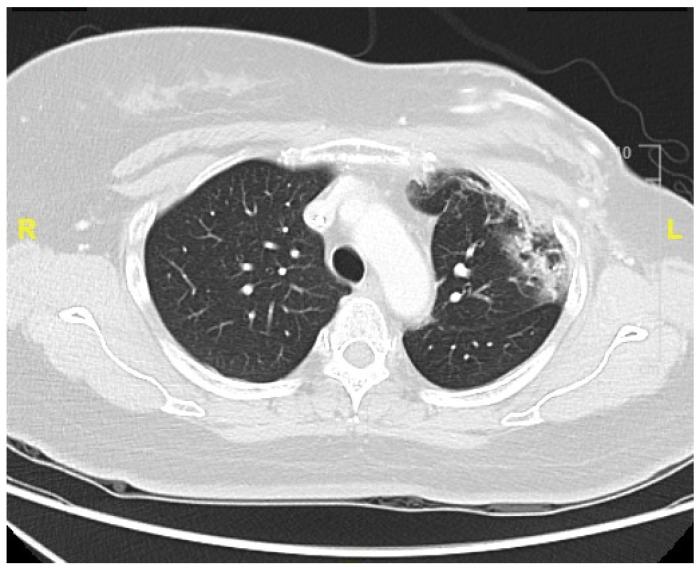
Chest computed tomography (CT) scan of lung window showed pneumonitis with subsegmental atelectasis, consolidation, and fibrotic changes in the left lung. (R: the right side. L: the left side.)

**Figure 3:**
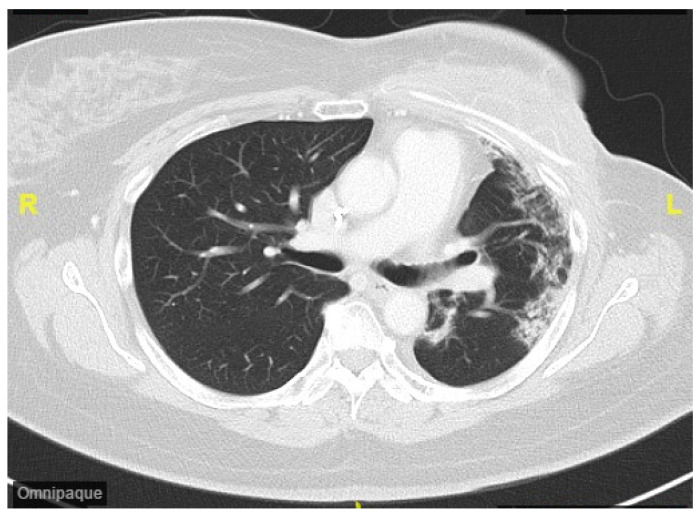
Chest CT of lung window showed prominent fibrosis, consolidation, and traction bronchiectasis in the left lung approximately 8 months after the completion of RT and initiation of abemaciclib. (R: the right side. L: the left side.)

**Figure 4:**
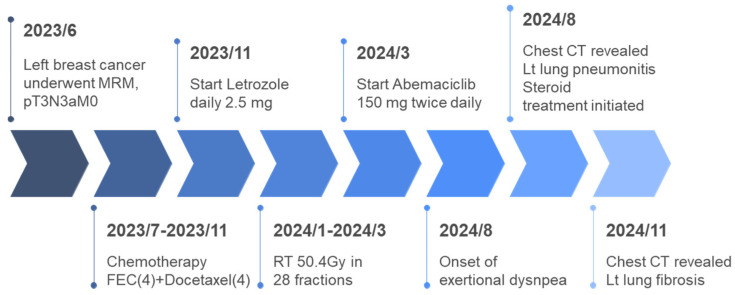
The timeline of breast cancer treatment and subsequent development of pneumonitis.

CDK4/6i abemaciclib (150 mg twice daily) was initiated after the completion of RT. Five months later, the patient presented with exertional dyspnea. A chest CT scan with and without intravenous contrast was performed. The image of the lung window revealed subsegmental atelectasis, consolidation, and fibrotic changes in the left upper lung and lower lung, with suspected radiation- and CDK4/6i-induced pneumonitis (Figure 2). The radiological changes observed on the chest CT were characteristic of radiation pneumonitis and corresponded with the irradiated area of the left chest wall. The timing of onset was consistent with the typical presentation of pneumonitis following cancer therapy. As the patient showed no signs of infection, common differential diagnoses such as pneumonia or respiratory tract infection were considered unlikely. The patient had no history of chronic obstructive pulmonary disease or heart failure, and cardiac function tests performed before and after cancer treatment demonstrated preserved ventricular function, thereby excluding the exacerbation of these comorbidities. During the follow-up period, there was no evidence of cancer recurrence, effectively ruling out lymphangitic carcinomatosis. Consequently, a diagnosis of therapy-related pneumonitis was established. The severity was graded as Grade 2 according to version 5 of the CTCAE. The spirometry test revealed a mild restrictive lung defect with a forced expiratory volume in one second (FEV_1_) of 1.68 L (88% of predicted) and a forced vital capacity (FVC) of 1.93 L (84% of predicted). Lung diffusion testing indicated a moderate impairment in the diffusion capacity for carbon monoxide (DLCO: 47% of predicted). Oral prednisolone therapy was initiated. At the three-month follow-up, a chest CT demonstrated prominent fibrosis, consolidation, and traction bronchiectasis in the left lung (Figure 3). The patient’s dyspnea improved after one month of oral betamethasone titration therapy. The positive response to corticosteroid therapy further supported the diagnosis of therapy-induced pneumonitis. The timeline of breast cancer treatment and the subsequent development of pneumonitis is listed in Figure 4.

## 4. Discussion

For patients with advanced breast cancer, treatment strategies include loco-regional therapies such as surgery and RT, as well as systemic therapies such as chemotherapy, targeted therapy, endocrine therapy, and immunotherapy. These novel cancer treatments have contributed to improved disease control and reduced mortality. This study reports a case of an Asian female with breast cancer who received a comprehensive post-operative treatment regimen, including chemotherapy, RT, endocrine therapy, and the recently approved CDK4/6i for curative adjuvant therapy. The patient developed treatment-related pneumonitis five months after receiving RT and abemaciclib.

For patients with advanced-stage disease or LN involvement, post-operative RT with RNI significantly reduces mortality and recurrence [15,16]. CDK4/6i combined with endocrine therapy has demonstrated PFS and OS benefit for HR-positive and HER2-negative advanced breast cancer, as confirmed by the PALOMA, MONALEESA, and MONARCH trials [1,2,3,4,5]. However, these treatments are associated with certain unavoidable side effects. As multimodal treatments become increasingly common in aggressive cancer management, clinicians must be cautious of unpredictable toxicity patterns and severity.

Radiation pneumonitis is a well-known concern following chest irradiation, with its incidence correlating with the lung radiation dose [17,18]. The other risk factors associated with the development of radiation pneumonitis include poor performance status, older age at diagnosis, smoking history, baseline pulmonary function, and the use of concurrent medications such as chemotherapy, immunotherapy, and target therapy. The dosimetric constraints recommended by the QUANTEC and ESTRO guidelines aim to minimize pulmonary toxicity, with suggested limits of a mean lung dose < 23 Gy and V20 < 35%. With the evolution of radiation techniques from three-dimensional conformal radiation therapy (3D-CRT) to modern modalities such as intensity-modulated radiotherapy (IMRT), volumetric modulated arc therapy (VMAT), and particle therapy, the ability to meet these constraints has improved, resulting in a reduced incidence of radiation pneumonitis. In the reported case, the radiation plan adhered to these constraints, with an ipsilateral lung mean dose of 11.9 Gy and V20 of 21.9%, and a whole lung mean dose of 7.5 Gy with V20 of 10.3%. The primary risk factor identified in this case was the use of concurrent medication. CT remains the primary imaging modality for the diagnosis of radiation pneumonitis. In the acute phase (within 1–6 months post-RT), typical radiological findings include ground–glass opacities (GGO) and consolidation localized to the irradiated lung regions. In the late phase (6–12 months post-RT), characteristic features include lung fibrosis, volume loss, consolidation, and traction bronchiectasis. In this case, CT imaging demonstrated findings consistent with the typical radiological features of both the acute phase (at 5 months after completion of RT) and late phase (at 8 months after RT). Furthermore, the patient’s good response to corticosteroid therapy supported the diagnosis of radiation pneumonitis.

While lung toxicity is relatively rare when CDK4/6i is used alone, ILD or pneumonitis is an uncommon but potentially severe adverse effect. The mechanism of CDK4/6i-induced ILD remains unclear. A preclinical study in mice suggested that palbociclib may augment inflammatory cell recruitment and enhance pulmonary inflammation [19]. Whether combining chest RT with CDK4/6i increases the risk of lung toxicity remains controversial [10,12,13,14,20,21]. Small case series have reported severe pneumonitis in patients receiving combination of CDK4/6i and chest RT, primarily in the context of metastatic breast cancer treated with low-dose palliative RT. Given the increasing use of CDK4/6i as a first-line treatment and their application in curative adjuvant settings, the potential for lung toxicity warrants further investigation.

The most common side effects of CDK4/6i are hematologic and GI toxicities [22]. The U.S. Food and Drug Administration (FDA) has issued warnings about the potential for drug-induced lung toxicity associated with CDK4/6i, as reported in the FDA Adverse Event Reporting System (FAERS). ILD appears to be more frequently associated with abemaciclib (2.1%) compared to palbociclib or ribociclib (0.3%) [23], although the overall incidence remains low [21,24,25,26]. Notably, FAERS data suggest that CDK4/6i-related ILD is more common in Asian populations, particularly among Japanese patients, accounting for nearly half of reported cases. In the monarchE trial, which evaluated the efficacy of abemaciclib as an adjuvant treatment, 95.4% of patients in both the abemaciclib arm and control arms had received prior RT. ILD occurred in 2.7% of patients in the abemaciclib arm and 1.2% in the control arm. Among different ethnic groups, ILD incidence was higher in Asian patients (6.6%) compared to the overall cohort (2.3%) [6,20,27]. A Japanese study reported an even higher incidence of CDK4/6i-induced ILD than previously documented in the PALOMA and MONARCH trials, with ILD occurring in 8.0% of patients (13% of abemaciclib-treated cases and 6.5% of palbociclib-treated cases), and a median onset time of approximately six months [20]. Given that our patient was an Asian female treated with abemaciclib, the risk of ILD may have been elevated.

Regarding other safety concerns associated with combining RT and CDK4/6i, case series and systematic reviews have reported GI and skin toxicities when these treatments are used either sequentially or concurrently. Notably, these toxic effects may persist for several months following treatment [10,14,28]. Currently, strong evidence or consensus on the toxicity of combining RT with CDK4/6i in the adjuvant setting remains lacking. It remains unclear whether temporary suspension of CDK4/6i during RT is necessary or whether concurrent or sequential administration is feasible. These issues warrant further investigation, as an increasing number of breast cancer patients will undergo multimodality treatments in the modern era.

## 5. Conclusions

A multimodality therapy approach is crucial in breast cancer treatment to maximize disease control and minimize therapy-related side effects. In breast cancer patients receiving a combination of RT and CDK4/6 inhibitors as curative adjuvant treatment, pulmonary toxicity is a concern and requires careful monitoring, particularly in Asian populations, even using modern RT techniques to limit the lung exposure dose.

## Data Availability

The data presented in this study are available on request from the corresponding author. The data are not publicly available due to patients’ privacy and medical ethics.
